# Next Generation Flow and Next Generation Sequencing for Measurable Residual Disease Assessment in Multiple Myeloma Patients: A Real‐Life Italian Multicenter Harmonization Experience

**DOI:** 10.1002/cam4.71678

**Published:** 2026-03-17

**Authors:** Stefania Oliva, Marina Martello, Elona Saraci, Silvia Armuzzi, Vincenza Solli, Simona Barbato, Angelo Belotti, Niccolò Bolli, Clara Bono, Francesco Buccisano, Marco Chiarini, Maria Antonietta Irno Consalvo, Iole Cordone, Daniela Drandi, Sara Galimberti, Elisa Genuardi, Viviana Giustini, Francesca Guerrini, Marta Lionetti, Akihiro Maheda, Sara Marino, Serena Masi, Antonio Matera, Andrea Mengarelli, Nunziatina Laura Parrinello, Aldo Maria Roccaro, Alessandra Romano, Giovanni Rossi, Barbara Taurisano, Alessia Tonini, Valentina Trimarco, Anna Maria Triolo, Ilaria Vigliotta, Renato Zambello, Benedetto Bruno, Michele Cavo, Elena Zamagni, Carolina Terragna

**Affiliations:** ^1^ Division of Hematology, Department of Molecular Biotechnology and Health Sciences University of Torino Turin Italy; ^2^ Division of Hematology, A O U Città Della Salute e Della Scienza di Torino University of Torino Turin Italy; ^3^ IRCCS Azienda Ospedaliero‐Universitaria di Bologna, Istituto di Ematologia “Seràgnoli” Bologna Italy; ^4^ Department of Medical and Surgical Sciences University of Bologna Bologna Italy; ^5^ Department of Hematology ASST Spedali Civili di Brescia Brescia Italy; ^6^ Department of Oncology and Hemato‐Oncology University of Milan Milan Italy; ^7^ Hematology Unit, Fondazione IRCCS Ca' Granda Ospedale Maggiore Policlinico Milan Italy; ^8^ Department of Clinical and Experimental Medicine University of Pisa Pisa Italy; ^9^ Hematology, Department of Biomedicine and Prevention University Tor Vergata Rome Italy; ^10^ Flow Cytometry Unit, Clinical Chemistry Laboratory, Diagnostic Department ASST Spedali Civili Brescia Italy; ^11^ IRCCS Regina Elena National Cancer Institute Clinical Pathology and Cancer Biobank and Haematology Unit Rome Italy; ^12^ A.O.U. Policlinico Rodolico‐San Marco U.O.C. Ematologia Catania Italy; ^13^ U.O.C di Ematologia e Trapianto di Cellule Staminali IRCCS Fondazione “Casa Sollievo Della Sofferenza” San Giovanni Rotondo Italy; ^14^ Department of Medicine (DIMED), Hematology Branch Padua University School of Medicine Padua Italy

**Keywords:** minimal residual disease, multiple myeloma, next‐generation flow‐cytometry, next‐generation sequencing, procedures harmonization

## Abstract

**Background:**

The level of measurable residual disease (MRD) is one of the most important features correlating depths of response and long‐term outcomes in multiple myeloma (MM) and MRD evaluation is currently the gold standard tool for assessing treatment response. Nevertheless, reproducibility across laboratories is a major concern, as discrepancies among results make comparability impractical. Aims: herein, we report preliminary results from the “Italian MM‐MRD network” project.

**Patients & Methods:**

MRD in bone marrow (BM) samples have been measured from newly diagnosed MM patients using next‐generation flow‐cytometry (NGF) or next‐generation sequencing (NGS) approaches in different laboratories.

**Results:**

The NGF workgroup (7 laboratories) implemented the Euro‐Flow Standard‐Operating‐Protocol to reach minimum 1 × 10^−5^ sensitivity. The inter‐operator retrospective study (Stage 1) showed high inter‐center concordance in monoclonal plasma cells detection (ICC = 0.90, *p* < 0.001), whereas moderate concordance was observed in the inter‐laboratory correlation (Stage 2) in in‐vivo samples (ICC = 0.63, *p* < 0.001), reaching a median limit‐of‐detection (LOD) and limit‐of‐quantification (LOQ) of 8 × 10−6 and 2 × 10^−5^, respectively. Greater variability was also observed in the analysis of other BM cell populations. The NGS workgroup (4 laboratories) employed a targeted amplicon‐based approach to detect clonotypic IGH/IGK gene rearrangements in diagnostic samples, subsequently used to track MRD in mock samples. The experimental design was divided into three quality‐control (QC) rounds, focused on finding a shared strategy for clonotype identification (QC1: 100% concordance among centers), or quantifying MRD in mock samples (concordance: 81% [QC2]; 91% [QC3]). The 10^−5^‐sensitivity level was successfully reached in most of tested dilutions (QC2: 19/20 = 95%; QC3: 19/23 = 83%).

**Conclusion:**

Overall, this pilot study provided preliminary data for MRD harmonization across Italian centers, paving the way for an expanded network, aiming at reducing variability, improving comparability, and enabling broader use of MRD‐monitoring in clinical practice.

## 1 Introduction

Multiple Myeloma (MM) is a neoplastic disorder characterized by the proliferation of malignant plasma cells (PCs) within the bone marrow (BM), more rarely in extramedullary sites. Although the therapeutic landscape has progressively expanded over the years, providing unprecedented deep and durable responses, MM remains incurable due to its heterogeneous nature and frequent recurrences. Indeed, despite achieving complete remission (CR), a small but clinically relevant population of MM cells—previously known as “minimal residual disease”, now named “measurable residual disease” (MRD)—may persist, leading to disease relapse. Therefore, MRD assessment has become crucial to monitor response to therapy, allowing predicting prognosis and risk stratification, and MRD assessment has been integrated into the new response criteria in MM [1].

Several techniques have been explored for MRD assessment, but only high‐throughput next‐generation sequencing (NGS) for molecular response evaluation and next‐generation flow‐cytometry (NGF) for evaluating residual PCs in the BM have been standardized [2]. In addition, the U.S. regulatory agency Food and Drug Administration (FDA) has recently recognized the value of MRD as a surrogate endpoint for predicting survival in MM and, therefore, for obtaining accelerated registration of new therapeutic combinations, making MRD evaluation through these methods routinely used in various clinical trials [3].

However, the use of different techniques can lead to discrepancies in results, making it infeasible to compare results obtained in different laboratories. This is, indeed, one of the main concerns in the implementation of MRD detection in clinical practice, underscoring the need to standardize MRD detection methods, according to the NGF/NGS criteria established by the International Myeloma Working Group (IMWG) [4]. To date, no prospective “real‐life” clinical trial with serial evaluations of MRD using both flow‐cytometry and molecular techniques has been conducted, and this remains an important gap.

The primary goal of the “Italian MM‐MRD Network” project has been to harmonize MRD analysis methods among the major hub hematology centers in Italy. Hopefully, this initiative will serve as a starting point to expand the network to other centers and extend the assessment of MRD in MM clinical practice by means of uniform and standardized techniques.

## 2 Methods

After an initial survey on the methods in use to detect MRD across various Italian hematology laboratories, 11 Italian laboratories with recognized experience in handling NGF and NGS techniques were selected to participate in this project, as further detailed below. Briefly, in this pilot study we decided to work in two separate groups (one for NGF and one for NGS) to avoid excessive bone marrow aspiration and consequently potential hemodilution of the samples, as well as to reduce the risk of potential shipping‐related issues. Indeed, in this preliminary study, our goal was to harmonize the two techniques separately, with the intention of converging in subsequent analyses and collaborative studies at a later stage. Collectively, a total of 20 adult patients with newly diagnosed MM (NDMM) were enrolled in this study and assessed for MRD between December 2021 and April 2024. All patients were treated as per standard care. Diagnostic samples were collected at the time of diagnosis (for NGS) and at first complete response or very good partial response (CR/VGPR) attainment and then shipped to the other laboratories for the analyses (details below). The Ethics Committee at each participating center approved the study. All patients provided written informed consent before entering the study, conducted in compliance with the Declaration of Helsinki and Good Clinical Practice guidelines.

### 2.1 Sample Collection (NGF)

Seven Italian hematology centers (L1: Brescia‐ASST Spedali Civili, L2: Catania‐AOU Policlinico, L3: Padova‐UniPd, L4: Roma‐IRCCS Regina Elena, L5: Roma‐Fondazione PTV, L6: S.Giovanni Rotondo‐IRCCS, L7: Torino‐UniTo), committed to adhering to the EuroFlow‐NGF protocol (www.euroflow.org), were selected to participate in the “NGF Harmonization Project”, coordinated by L7.

In the preliminary phase of the study (Stage 1), the inter‐operator variability was assessed. For this purpose, four laboratories (L1‐3, L7) performed blinded cytofluorimetric analysis of six anonymous files from a MM patient data repository provided by L7. In the subsequent phase (Stage 2), the inter‐laboratory variability of MRD quantification was evaluated using BM samples from 12 NDMM patients: 3 from L7 and L3, 2 from L1 and L2, 1 from L4 and L6. These patients underwent four cycles of Dara‐VTD (Daratumumab, bortezomib, thalidomide, and dexamethasone) induction therapy followed by autologous stem cell transplantation (ASCT), achieving CR or VGPR by day 100 (±15 days) post‐ASCT. BM aspirates were collected in a 20 mL syringe containing 1 mL of sodium citrate as an anticoagulant. The anonymized samples were then distributed equally into EDTA‐containing tubes and shipped at room temperature to all participating laboratories for simultaneous analysis, to be performed within 24–48 h from blood collection. MRD was evaluated according to NGF‐EuroFlow protocol [5], as described in Supporting Information.

### 2.2 Sample Collection (NGS)

Molecular MRD assessment by NGS involved 4 Italian “start‐up” centers (C1: Bologna‐IRCCS AOUBO, C2: Milano‐Fondazione IRCCS Ca'Granda, C3: Pisa‐AOU, C4: Torino‐UniTo), coordinated by C1.

Two specific quality control (QC) rounds related to the ID screening phase for clonotype identification (QC1) and the MRD assessment (QC2 and QC3) were performed. A total of 8 NDMM patients were enrolled, 4 from Bologna and 4 from Milan. In addition, 4 healthy donor PB samples were employed to prepare mock samples as controls. NGS, for ID clonotype definition and MRD measurements, was performed using LymphoTrack Dx IGH FR1/FR2/FR3 and IGK (Invivoscribe) assays [6, 7, 8, 9] on the Miseq Illumina platform. As part of QC1, participating centers were required to collect all detected rearrangement(s) for each patient, their frequencies, and score them according to their uniqueness. Under QC2 and QC3 rounds, the MRD results and LOQ of the experiment were collected.

C1 and C2 centers were responsible for preparing both DNA and cell fractions derived from the patients included in the study. Samples were treated as described in Supporting Information.

All metric results obtained from each experiment (i.e., pool quantification, cluster density, total reads, percentage of passing filter reads, reads > Q30) were collected, compared, and collegially discussed to delineate the most appropriate way to perform and report NGS screening results, both for clonotype(s) assessment and MRD measurements (Table S1). The clonotype(s) for IGH and IGK assays were defined according to the algorithms issued by Invivoscribe; in addition, the level of similarity between the IGH/IGK rearrangements and the immunoglobulin sequences deposited in IMGT (www.imgt.org; V‐QUEST database) [10] and NCBI (BLAST–Basic Local Alignment tool) [11] databases were checked. MRD was then calculated as a fraction of the total amount of analyzed cells, normalized against an internal control sample. The LOD was 10^−5^ and the LOQ was calculated from the effective number of total cells used as input for each reaction. Results of the MRD experiments were considered evaluable when each replicate reached at least 200.000 reads.

## 3 Results

### 3.1 NGF Workgroup Results

**FIGURE 1:**
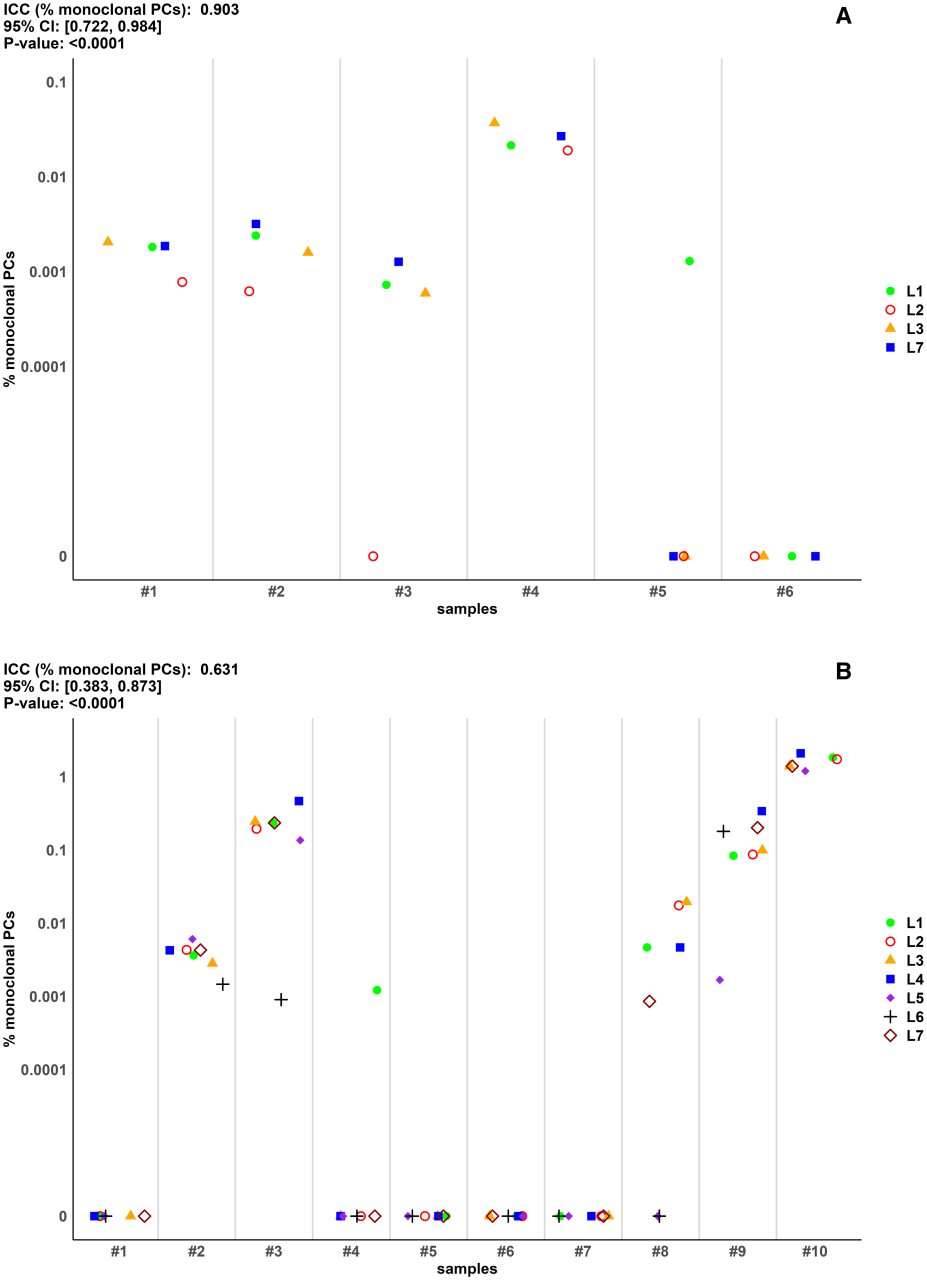
Concordance of NGF samples in study Stage 1 (1A) and Stage 2 (1B). L1‐L7 are the seven Italian laboratories selected for NGF analyses of minimal residual disease (L1: Brescia‐ASST Spedali Civili, L2: Catania‐AOU Policlinico, L3: Padova‐UniPd, L4: Roma‐IRCCS Regina Elena, L5: Roma‐Fondazione PTV, L6: S.Giovanni Rotondo‐IRCCS, L7: Torino‐UniTo). CI, confidence interval; ICC, intraclass correlation coefficient, PCs, plasma cells.

In Stage 1 of the study, 6 anonymized data files of MM patients were analyzed to evaluate inter‐operator variability. The task was to determine the MRD status of each sample based on the percentage of monoclonal mPCs. Overall, 100% of practitioners rated Samples #1, #2, and #4 as MRD‐positive, and Sample #6 as negative. Sample #3 was rated MRD‐positive by 75% of the participants, and 75% rated Sample #5 as MRD‐negative (ICC = 0.90, 95% CI 0.72–0.98, *p* < 0.001) (Figure 1A).

The variability in BM cell populations was also assessed, including the percentage of: lymphocytes (CD45++/SSC low); monocytes (CD38+/CD45++/SSC intermediate); neutrophils (CD45+/SSC high); erythroblasts (CD45−/CD138−); mast cells (CD117+++); regenerating B lymphocytes (CD19+/CD38+/CD81 high/CD45 low); mature B lymphocytes (CD19+/CD38‐/CD45++). The percentage of lymphocytes showed low variability and strong homogeneity between measurements, the same for neutrophils and monocytes. However, other cell populations, such as mast cells, erythroblasts, regenerating B lymphocytes, and mature B lymphocytes, showed greater variability, suggesting potential challenges in evaluating cell populations that are less prevalent in BM, but whose presence is substantial for assessing hemodilution (Table S2). Significant variability was observed in the measurement of the percentage of normal PCs (ICC = 0.30, 95% CI −0.061 < ICC < 0.807, *p* = 0.058). Also, a discrepancy was observed in the number of total events acquired and, consequently, in the LOD and LOQ values (Table S2). This was particularly evident in L2, as the parameters of tube 1 and tube 2 were not perfectly matched, and the Kaluza 2.1 software does not support merging the two data files.

In Stage 2, a total of 12 samples from as many NDMM patients were distributed and processed within 48 h of specimen collection to evaluate inter‐laboratory correlation. Two samples were excluded as considered “unassessable” due to the high percentage of cell mortality. Collectively, all laboratories agreed that Samples #2, #3, #9, and #10 were MRD‐positive. Similarly, all agreed that Samples #1, #5, #6, and #7 were MRD‐negative. Conversely, Sample #4 was considered MRD‐negative by 86% of participants, and Sample #8 was MRD‐positive by 71% (ICC = 0.63, 95% CI 0.38–0.87, *p* < 0.001) (Figure 1B).

Moderate concordance was observed for the detection of the percentage of normal PCs (ICC = 0.60, 95% CI 0.348–0.858, *p* < 0.001). However, significant discrepancies were found in the total number of acquired events, thereby resulting in variations in LOD and LOQ (Table S3). These discrepancies were discussed and mainly attributed to variability in the total volume and cellularity of distributed samples, or the final viability of white blood cells following sample transport. Overall, median LOD was 8 × 10^−6^ (range 2 × 10^−6^—6 × 10^−5^), and median LOQ was 2 × 10^−5^ (range 5 × 10^−6^ 1 × 10^−4^).

Notably, the greatest differences in antigen expression of monoclonal PCs were observed for CD45, CD27, and CD81. As in Stage 1, the variability of BM cell populations was assessed. Again, the percentage of lymphocytes showed low variability and greater homogeneity, similar to neutrophils and monocytes. Other populations, such as mast cells, erythroblasts, regenerating B lymphocytes, and mature B lymphocytes, showed greater variability (Table S3).

### 3.2 NGS Workgroup Results

#### 3.2.1 Quality Control 1: Clonotype Definition

QC1 was aimed at comparing performances of participating laboratories in the definition of patient‐specific clonotype(s) in the diagnostic samples. Each center successfully performed a V2 500 NGS experiment by loading 4 different assays for each patient, for a total of 37 reactions, including one positive and one negative control for both IGH and IGK assays, and a non‐template control. Overall, technical parameters were quite similar among centers: cluster density was around 1000 k/mm^2^ (range 910–1090) with a median of 93.2% of reads passing filters (range 83.7–96.7) and 89.1% of reads > Q30 (range 86–93). Moreover, the IGH positive control top percentage reads were consistently ≥ 2.5%, whereas the negative control top percentage reads were consistently < 1 (Table 1).

**TABLE 1:** Quality‐control round 1 (QC1): Clonotype identification by next‐generation sequencing (NGS).

QC1		C1			C2			C3			C4		
PT	Assay	Identified clonotype	% Merged reads	Clonotype to track	Identified clonotype	% Merged reads	Clonotype to track	Identified clonotype	% Merged reads	Clonotype to track	identified clonotype	% Merged reads	Clonotype to track
**PT_1**	**IGHFR1**	**IGHV5‐51_01 IGHJ4_02**	**90.96**	**IGHFR1**	IGHV5‐51_01‐J4‐02	83.90%	**IGHFR2**	IGHV5‐51/J4‐02	88.78%	**IGHFR3/IGK**	**IGHV5‐J4**	**79.9**	**IGHFR1/IGHFR2/IGHFR3/IGK**
	IGHFR2	IGHV5‐51_01 IGHJ4_02	88.87		**IGHV5‐51_01‐J4‐02**	**87.94%**		IGHV5‐51/J4‐02	72.61%		**IGHV5‐J4**	**91.9**	
	IGHFR3	IGHV5‐51_02 IGHJ4_02	84.23		(< 10,000 tot reads)			**IGHV5‐51/J4‐02**	**92.21%**		**IGHV5‐J4**	**85.8**	
	IGK	IGKV2D‐26_01 IGKJ4_01	37.30		IGKV2D‐26_01‐J4_01	34.77%		**IGHV2D26/JH4**	**34.01%**		**IGKV2D/J4**	**36.9**	
**PT_2**	IGHFR1	IGHV3‐33_03 IGHJ6_02	91.76	**IGHFR3**	IGHV3‐33_03‐IGHJ6_02	86.10%	**IGHFR3**	IGHV3‐33/J6	92.36%	**IGHFR3/IGK**	**IGHV3‐J6**	**82.6**	**IGHFR1/IGHFR2/IGHFR3/IGK**
	IGHFR2	IGHV3‐33_03 IGHJ6_02	86.40		IGHV3‐33_03‐IGHJ6_02	81.28%		IGHV3‐33/J6	85.18%		**IGHV3‐J6**	**83.1**	
	**IGHFR3**	**IGHV4‐39_02 IGHJ6_02**	**97.34**		**IGHV4‐39_02‐IGHJ6_02**	**98.67%**		**IGHV3‐39/J6**	**98.63%**		**IGHV4‐J6**	**97.4**	
	IGK	IGKV1‐9_01 IGKJ2_03	24.31		IGKV3‐11_01‐IGKDEL	57.62%		**IGKV3‐11/DEL V1‐9/J2**	**54.64% 38.37%**		**IGK3/IGKdel**	**49.9**	
**PT_3**	**IGHFR1**	**IGHV4‐61_01 IGHJ5_02**	**88.45**	**IGHFR1**	IGHV4‐61_01‐IGHJ5_02	86.62%	**IGHFR3**	IGHV4‐61/J5	91.62%	**IGHFR3/IGK**	**IGHV4‐J5**	**82.9**	**IGHFR1/IGHFR2/IGHFR3/IGK**
	IGHFR2	IGHV4‐61_01 IGHJ5_02	89.96		IGHV4‐61_07‐IGHJ5_02	82.52%		IGHV4‐61/J5	76.66%		**IGHV4‐J5**	**86.8**	
	IGHFR3	IGHV4‐61_07 IGHJ5_02	99.51		**IGHV4‐61_07‐IGHJ5_02**	**99.24%**		**IGHV4‐61/J5**	**99.07%**		**IGHV4‐J5**	**98.2**	
	IGK	IGKV3‐15_01 IGKDEL	93.68		IGKV3‐15_01‐IGKDEL	92.58%		**IGKV315/DEL**	**94.45%**		**IGK3/IGKdel**	**90.7**	
**PT_4**	**IGHFR1**	**IGHV3‐43_01 IGHJ2_01**	**88.52**	**IGHFR1**	**IGHV3‐43_01‐IGHJ2_01**	**84.38%**	**IGK**	**IGHV3‐43/J2**	**91.06%**	**IGHFR1/IGK**	**IGHV3‐J2**	**75.9**	**IGHFR1/IGHFR2/IGK**
	IGHFR2	IGHV3‐43_01 IGHJ2_01	8.77		(< 10,000 tot reads)			No clonality			**IGHV3‐J2**	**9.5**	
	IGHFR3	POLI	POLI		(< 10,000 tot reads)			No clonality			(< 10,000 tot reads)		
	IGK	IGKV4‐1_01 IGKJ4_01	93.79		IGKV4‐1_01‐IGKJ4_01	97.70%		**IGKV4‐01/J4**	**97.54%**		**IGK4/J4**	**95.9**	
**PT_5**	IGHFR1	IGHV1‐8_02 IGHJ4_02	45.35	**IGHFR3**	IGHV1‐8_02‐IGHJ4_02	50.39%	**IGHFR1**	IGHV1‐08/J4	44.50% (non prod) 79.25 (prod)	**IGHFR1/IGK**	**IGHV1‐J4**	**65.5**	**IGHFR1/IGHFR3/IGK**
	IGHFR2	POLI	POLI		(< 10,000 tot reads)			No clonality			(< 10,000 tot reads)	< 2.5%	
	**IGHFR3**	**IGHV1‐f_01 IGHJ4_02**	**57.29**		(< 10,000 tot reads)			IGHV1‐01/J4	62.94% (non prod) 31.38 (prod)		**IGHV1‐J4**	**92.5**	
	IGK	IGKV3D‐20_01‐J1_01	94.95		(< 10,000 tot reads)			**IGKV3D20–01/J1**	**89.12%**		**IGK3/J1**	**87.8**	
**PT_6**	IGHFR1	IGHV4‐59_01 IGHJ4_02	3.53	**IGHFR3**	(< 10,000 tot reads)		**IGHFR3**	IGHV4‐59/J4	2.72%	**IGHFR3/IGK**	(< 10,000 tot reads)		**IGHFR3/IGK**
	IGHFR2	POLI	POLI		(< 10,000 tot reads)			IGHV4‐61/J4	2.60%		(< 10,000 tot reads)		
	**IGHFR3**	**IGHV4/OR15‐8_03 IGHJ4_02**	**97.42**		**IGHV4/OR15‐8_03‐IGHJ4_02**	**98.38%**		IGHV1‐01/J4	62.94 (non prod) 31.38 (prod)		**IGHV4‐J4**	**96.6**	
	IGK	IGKV5‐2_01 NONE	58.26		IGKV5‐2_01‐none	68.40%		**IGKV5/none INTR/DEL**	**60.59% 20.23%**		**IGK5/none**	**73.3**	
**PT_7**	IGHFR1	IGHV3‐15_02 IGHJ4_02	88.50	**IGHFR2**	IGHV3‐15_02‐IGHJ4_02	86.41%	**IGK**	IGHV3‐15/J4	90.67%	**IGHFR1/IGK**	**IGHV3‐J4**	**82**	**IGHFR1/IGHFR2/IGHFR3**
	**IGHFR2**	**IGHV3‐15_02 IGHJ4_02**	**82.07**		IGHV3‐15_02‐IGHJ4_02	85.54%		IGHV3‐15/J4	88.01%		**IGHV3‐J4**	**88.5**	
	IGHFR3	IGHV3‐15_07 IGHJ4_02	67.66		(< 10,000 tot reads)			IGHV3‐15/J4	84.69%		**IGHV3‐J4**	**90.4**	
	IGK	IGKV4‐1_01 IGKJ2_01	63.05		**IGKV4‐1_01‐IGKJ2_01**	**89.38%**		**IGKV4‐01/J2 V311‐J2**	**74.54% 23.41%**		(< 10,000 tot reads)	no INTR‐kde	
**PT_8**	**IGHFR1**	**IGHV3‐7_03 IGHJ4_02**	**90.90**	**IGHFR1**	IGHV3‐7_03‐IGHJ4_02	88.87%	**IGHFR2**	**IGHV3‐7/J4**	**93.23%**	**IGHFR1/IGK**	**IGHV3‐J4**	**80.8**	**IGHFR1/IGHFR2/IGK**
	IGHFR2	IGHV3‐7_03 IGHJ4_02	79.67		**IGHV3‐7_03‐IGHJ4_02**	**90.89%**		IGHV3‐7/J4	89.39%		**IGHV3‐J4**	**87.9**	
	IGHFR3	IGHV7‐4‐1_04 IGHJ6_03	20.41		(< 10,000 tot reads)			No clonality			(< 10,000 tot reads)		
	IGK	IGKV4‐1_01‐J2_01	41.95		IGKV4‐1_01‐IGKJ2_01	48.55%		**IGKV4‐01/J2 V1‐9/J4 INTR/DEL**	**45.50% 21.23% 16.81%**		**IGK4/J2**	**49.5**	
**CTR_IGH_POS**	**IGHFR3**	IGHV1J4	2.15		IGHV1‐46/J4	2.22		IGHV1‐46/J4	2.22		VH1/J4	2.6	
**CTR_IGH_NEG**	**IGHFR3**	IGHV2‐70/J4	0.09		IGHV2‐70/J4	1.20		IGHV2‐70/J4	0.07		< 10,000 reads		
**CTR_IGK_POS**	**IGK**	IGK V3D_DEL	19.00		IGKV3D‐20_01‐IGKDEL	13.39%		IGKV3‐20/DEL	11.12		V3D/del	12.1	
**CTR_IGK_NEG**	**IGK**	IGKV3D20–01/J1	1.18		(< 10,000 tot reads)			IGKV3D20–01/J1	1.11		< 10,000 reads		

*Note:* Results from the first NGS experiment performed by the network of the 4 laboratories (C1, C2, C3, and C4) (Table S1). Results of the first QC1. Centres performed NGS experiments on 8 different patients (Pt) by using 4 different assays: IGH FR1, FR2, FR3, and IGK. Both the identified clonotypes and the number of merged reads has been reported. Additional evaluations of clonotype characteristics have been checked on IMGT database and Blast and have been reported in Table S4. Bold values indicate the trackable clonotypes identified by each center and either confirmed or not in the “clonotype to track” column.

NGS experiments were designed to obtain ≥ 200.000 reads for each reaction, to guarantee clonality detection with enough confidence. Even though some reactions failed in two participating centers (11/36 and 6/36, in C2 and C4, respectively), the overall rate of success in the clonotype identification was approximately 88%.

The criteria for defining clonality of IGH/IGK rearrangements were: ≥ 1 rearrangement on the top merged reads (at least ≥ 2.5% of total reads) and its percentage of merged reads at least doubled compared with the third most abundant rearrangement on top merged reads. Additionally, the originality of rearrangements was also verified through comparison with the sequences included in the V‐quest immunoglobulin database and the BlastN database; in case of ambiguity, the identity of V‐ and J‐genes, the number of mismatched nucleotides in the junction region, and the BlastN score were considered as additional criteria to infer sequence originality (Table 1; Figure S2 and Table S4).

Collectively, QC1 demonstrated 100% concordant identification of rearrangements by NGS across centers, considering only successful experiments. Major discussions emerged around selecting the trackable clonotype, especially when multiple regions (such as IGHFR1, FR2, FR3, and IGK) were suitable for this purpose. Due to the lack of clear guidelines for clonotype selection to monitor MRD, agreement among centers varies widely (from 50% to 100%), highlighting the need for standardized, shared recommendations.

#### 3.2.2 Quality Control 2–3: MRD Assessment

QC2 and QC3 were focused on MRD measurement, starting from shared DNA or cells mock samples. To this aim, centers were asked to perform three V3 600 runs on Miseq and to test the clonotype assay selected in QC1. Each experiment was designed to include a maximum of 19 reactions: for each of the 8 patients included in the study, both MRD mock samples (10^−3^ and 10^−4^ dilutions) were tested in three replicates. These experimental conditions were supposed to allow the identification of the tracked clone with enough sensitivity, starting from 2 μg of DNA and producing at least 2 million reads per replicate. In the QC2, centers were asked to perform the NGS experiments starting directly from the DNA provided by C1 and C2 centers; even though some library preparations failures were observed (up to 9 of failed reactions out of 48 total reactions per center, success rate ranging from 81.2% to 100%: C1 = 0, C2 = 9, C3 = 3) (Table 2), all centers have been able to detect the tracked clone with an overall concordance rate of 81%, main discordances deriving from the level of confidence used to detect the tracked clone. In the QC3, centers were asked to perform the NGS experiments starting from the aliquot of cells provided by C1 and C2 centers. This additional DNA extraction was planned to evaluate whether different genomic isolation methods might impact on sequencing results, and to evaluate which starting materials (either cells or DNA) would be better for quality control when sharing samples in the future. The overall concordance rate of MRD measurements was 91%, with lower number of failed libraries (up to 3 of failed reactions out of 48 total reactions per center, success rate ranging from 93.7% to 100%; C1 = 0, C2 = 3, C3 = 0) (Table 3). Moreover, the level of sensitivity of 10^−5^ has been successfully reached for each patient in most of the tested dilutions (QC2: 19/20 = 95%, QC3: 19/23 = 83%), again supporting the robustness of the method.

**TABLE 2:** Quality‐control round 2 (QC2): MRD monitoring starting from DNA by next‐generation sequencing (NGS).

QC2‐DNA		Centres	Sequence	MRD results	Clonal frequency	Concordance			
						Dilution	N. concordant results	%	Overall QC2 concordance %
**PT_1**	**DIL1**	**C1**	**IGHV5‐JH4**	**Detected**	**2.46E‐03**	**DIL1**	**2/2**	**100**	**81**
	**DIL2**			**Detected**	**1.87E‐04**				
	**DIL1**	**C2**	**Lib prep failed**	NE	NE				
	**DIL2**			NE	NE	**DIL2**	NE	NE	
	**DIL1**	**C3**	**IGHV5‐JH4**	**Detected**	**1.39E‐03**				
	**DIL2**			NE	NE				
**PT_2**	**DIL1**	**C1**	**IGHV4‐JH6**	**Detected**	**2.01E‐02**	**DIL1**	**3/3**	**100**	
	**DIL2**			**Detected**	**2.25E‐03**				
	**DIL1**	**C2**	**IGHV4‐JH6**	**Detected**	**2.39E‐02**				
	**DIL2**		**Lib prep failed**	NE	NE	**DIL2**	**2/2**	**100**	
	**DIL1**	**C3**	**IGHV4‐JH6**	**Detected**	**6.01E‐02**				
	**DIL2**			**Detected**	**2.03E‐03**				
**PT_5**	**DIL1**	**C1**	**IGHV1‐JH4**	**Detected**	**1.51E‐04**	**DIL1**	**2/3**	**67**	
	**DIL2**			**Detected**	**1.61E‐05**				
	**DIL1**	**C2**	**IGHV1‐JH4**	**Detected**	**2.86E‐05**				
	**DIL2**			**Detected**	**2.87E‐06**	**DIL2**	**2/3**	**67**	
	**DIL1**	**C3**	**IGHV1‐JH4**	**Detected**	**1.75E‐04**				
	**DIL2**			**Detected**	**1.75E‐06**				
**PT_6**	**DIL1**	**C1**	**IGHV4‐JH4**	**Detected**	**1.22E‐02**	**DIL1**	**2/3**	**67**	
	**DIL2**			**Detected**	**6.27E‐04**				
	**DIL1**	**C2**	**IGHV4‐JH4**	**Detected**	**9.66E‐03**				
	**DIL2**			**Detected**	**1.33E‐03**	**DIL2**	**2/3**	**67**	
	**DIL1**	**C3**	**IGHV4‐JH4**	**Detected**	**1.06E‐02**				
	**DIL2**			**Detected**	**9.83E‐04**				

*Note:* Results from the first NGS experiment performed by the network of 3 laboratories (C1, C2, and C3). For each patient (Pt) included in the QC2, both MRD mock sample dilutions (DIL1 and DIL2) have been reported. Concordance has been assessed by comparing, across centers, both the MRD results and the Clonal frequency for each evaluable dilution, using C1 as the reference center. Cells were highlighted in green when results were concordant and in red when they were discordant. Not evaluable results (NE) were not included in concordance evaluation and left blank. A QC2 overall concordance rate has been calculated through a mean of all the concordant results obtained from each dilution between centres. Bold values do not have any specific significance, as they are distributed throughout the tables.

**TABLE 3:** Quality‐control round 3 (QC3): MRD monitoring starting from CELLS by NGS.

QC3‐cells		Sequence	MRD results	Clonal frequency	Concordance			
					Dilution	N. concordant results	%	Overall QC3 concordance %
**PT_3**	**DIL1**	**IGHV4‐JH5**	**Detected**	**6.26E‐03**	**DIL1**	**2/2**	**100**	**91**
	**DIL2**		**Detected**	**8.09E‐04**				
	**DIL1**	**IGHV4‐JH5**	**Detected**	**6.83E‐03**				
	**DIL2**		**Detected**	**4.70E‐04**	**DIL2**	**3/3**	**100**	
	**DIL1**	**IGHV4‐JH5**	NE	NE				
	**DIL2**		**Detected**	**4.00E‐04**				
**PT_4**	**DIL1**	**IGHV3‐JH2**	**Detected**	**8.16E‐03**	**DIL1**	**2/3**	**67**	
	**DIL2**		**Detected**	**6.04E‐04**				
	**DIL1**	**IGHV3‐JH2**	**Detected**	**1.34E‐03**				
	**DIL2**		**Detected**	**2.08E‐04**	**DIL2**	**3/3**	**100**	
	**DIL1**	**IGHV3‐JH2**	**Detected**	**4.00E‐04**				
	**DIL2**		**Detected**	**1.56E‐04**				
**PT_7**	**DIL1**	**IGHV3‐JH4**	**Detected**	**2.23E‐03**	**DIL1**	**3/3**	**67**	
	**DIL2**		**Detected**	**2.86E‐04**				
	**DIL1**	**IGHV3‐JH4**	**Detected**	**3.40E‐03**				
	**DIL2**		**Detected**	**2.05E‐04**	**DIL2**	**2/3**	**67**	
	**DIL1**	**IGHV3‐JH4**	**Detected**	**1.34E‐03**				
	**DIL2**		**Detected**	**7.65E‐05**				
**PT_8**	**DIL1**	**IGHV3‐JH4**	**Detected**	**5.37E‐03**	**DIL1**	**3/3**	**67**	
	**DIL2**		**Detected**	**6.84E‐04**				
	**DIL1**	**IGHV3‐JH4**	**Detected**	**5.52E‐03**				
	**DIL2**		**Detected**	**8.06E‐04**	**DIL2**	**3/3**	**67**	
	**DIL1**	**IGHV3‐JH4**	**Detected**	**4.05E‐03**				
	**DIL2**		**Detected**	**1.85E‐04**				

*Note:* Results from the first NGS experiment performed by the network of 3 laboratories (C1, C2, and C3). For each patient (Pt) included in the QC3, both MRD mock sample dilutions (DIL1 and DIL2) have been reported. Concordance has been assessed by comparing, across centers, both the MRD RESULTS and the CLONAL FREQUENCY for each evaluable dilution, using C1 as the reference center. Cells were highlighted in green when results were concordant and in red when they were discordant. Not evaluable results (NE) were not included in concordance evaluation and left blank. A QC3 overall concordance rate has been calculated through a media of all the concordant results obtained from each dilution between centres. Bold values do not have any specific significance, as they are distributed throughout the tables.

Overall, the results obtained in QC2 and QC3 showed that sharing cells, rather than DNA, provided more reliable results for MRD evaluation. Moreover, although the MRD levels of the mock samples were 10^−3^ and 10^−4^, the minimum level of sensitivity of 10^−5^ required to support clinical decisions has been successfully reached by most of the participating centers.

## 4 Discussion

Recent data and meta‐analyses have shown that MRD has a significant impact on both progression‐free survival and overall survival in various cohorts of MM patients [12, 13, 14, 15, 16, 17], regardless of cytogenetic risk, assessment method, or conventional response, showing clinical significance in both standard‐risk and high‐risk patients. Currently, MRD assessment is widely used in clinical studies as an early indicator of treatment efficacy and is being increasingly integrated into the clinical practice of many hematology centers [18, 19, 20, 21], as accurate determination of MRD allows monitoring response to therapy, predicting prognosis, and risk stratification.

Considering these advancements, during a meeting of the Oncologic Drugs Advisory Committee (ODAC) held by the U.S. FDA (Docket No. FDA‐2024‐N‐1179), panelists voted unanimously in favor of using MRD as an early endpoint in clinical trials, supporting its potential for accelerated approval of therapies in MM. MRD is therefore expected to play a key role in therapeutic decision‐making and treatment personalization soon, aiming at improving clinical outcomes by intensifying or de‐intensifying treatment schedules, or serving as an early marker of disease relapse. Despite substantial evidence and endorsement by regulatory bodies supporting the prognostic value of MRD, significant variability in its implementation remains, as recently highlighted by three Delphy surveys [22, 23, 24]. Results of these surveys conducted in US, Europe and India showed significant global variability in MRD testing with respect to methodology, thresholds, use of functional imaging, and the clinical management of MRD‐positive patients. Barriers to the routine implementation of MRD assessment in clinical practice include the lack of universal adoption of MRD testing, the absence of standardized protocols recommending its use, and limited accessibility. Thereby, the implementation of methodologies to measure MRD and, more importantly, standardized procedures to harmonize results among different laboratories, are matters of utmost importance and have been recently highlighted by three different and independent Delphi methods' surveys [22, 23, 24].

In this regard, both NGF and NGS are highly sensitive techniques, each with distinct strengths and limitations. NGF is particularly advantageous at key clinical decision‐making time points due to its rapid turnaround, broad applicability, high sensitivity, and standardized implementation, enabling timely therapeutic adjustments and facilitating its integration into routine clinical practice. In contrast, NGS may offer added value in selected clinical scenarios, including baseline clonal characterization, longitudinal tracking of disease evolution, and the assessment of MRD in patients achieving deep and sustained responses, where its higher analytical sensitivity may improve risk stratification. However, its wider use may be constrained by higher costs, longer processing times, and the requirement for baseline diagnostic samples. Therefore, the choice between NGF and NGS should be tailored to the clinical setting and specific study aims.

Nevertheless, despite these differences, both techniques have demonstrated strong biological and clinical correlation, with nearly identical prognostic value [2, 25, 26]. Providing a high level of expertise, both techniques can be efficiently used (as long as the EuroFlow guidelines [27] are adopted for NGF), allowing centers to choose depending on experience, feasibility, and cost considerations. Nonetheless, comparability of results obtained by different techniques or laboratories is a major concern. In the absence of ad‐hoc prospective trials designed to compare serial MRD evaluation across various centers, we conducted a pilot study aimed at creating a national MRD‐MM‐network among 11 hematology centers with solid expertise in the field of these next‐generation techniques, with the goal of sharing methods and harmonizing MRD evaluation.

Overall, our study demonstrated a high level of agreement (92% in Stage 1; 96% in Stage 2) in MRD identification (negativity vs. positivity) by NGF. On the other hand, the correlation coefficient was higher in Stage 1 (ICC: 0.9) vs. Stage 2 (ICC: 0.63); this can be explained by a higher inter‐laboratory variability in the report of percentage of MRD results, likely attributable to the impact of transportation on cell viability and/or challenges in sample processing as well as the use of different software in some laboratories for the analysis of data files, compared to the inter‐operator study which was a retrospective analysis on samples processed by a single laboratory. It should be noted, however, that discrepancies were already evident during this preliminary phase, highlighting that structured training and operator expertise are critical determinants of reliable MRD assessment; these factors, together with software compatibility, likely contributed to the observed inter‐operator variability.

However, following SOP guidelines for sample processing and staining, standardization of instrument settings, and harmonization of assay protocols using a well specified NGF KIT for PCs antigen expression has granted a high degree of reproducible MRD results (Figure 1A,B), confirming previous results on 20 analyzed samples in a European Myeloma Network study reaching 95% of concordant cases [28].

The major concern outlined here was the number of acquired events in the samples, which translated into greater variability of LOD and LOQ values. This was mainly related to the variability of total volume and, consequently, cellularity of the distributed samples and transport conditions, as previously mentioned. Moreover, the timing of performing analysis was confirmed to be critical for maintaining viability and thus evaluability of the sample; indeed, processing samples after 48 h from BM aspiration resulted in high mortality, underscoring the importance of improving the logistics of shipping samples between different laboratories, or of centralizing analysis, and the need for shared guidelines and well specified tubes and fluorochromes.

Notably, low variability and greater consistency in the measurements of lymphocytes, neutrophils, and monocytes were noted in both study stages. By contrast, other populations such as mast cells, erythroblasts, regenerating B lymphocytes, and mature B lymphocytes, which are present in small percentages in the BM compartment and typically under‐represented by flow‐cytometry, showed more discordant results, particularly in Stage 2 (e.g., mast cells). Therefore, collecting a high number of events is recommended to ensure adequate analytical sensitivity and select these cell populations properly, as they are crucial for assessing the hemodilution of the sample [4]. In addition, as reported by Soh et al. [29], the explicit definition of gating during the creation of the draft consensus analysis strategy led to greater reproducibility of mast cells and hematogones enumeration and determination of sample adequacy cut‐off values for all marrow constituents. Furthermore, we agree with the authors that, to achieve greater consistency, the use of software, as also recommended by EuroFlow protocol, as infinicyt, could help to handle data in a homogeneous manner, especially for calculating “Total Number of Analyzed Cells,” “Total PCs,” “Abnormal PCs,” and “Hematogones.” The ability to combine automatically FCS data from both tubes in the final report, with the advantage of increasing the total number of abnormal PCs detected, which—when properly validated—amplifies the LOD and LOQ of the flow assay becomes essential to avoid false negatives (Sample #3 Figure 1A).

Another point of strategic relevance, in our opinion, is the way to report the flow‐MRD study. A previous Italian regional multicenter study focused on the data to be included in the clinical report of MRD testing in MM. By ensuring the operational autonomy of each laboratory, a uniform and shared report was proposed to provide physicians with a clear, complete report that limits subjective interpretations, highlights possible limitations of the study, and better supports clinical decision‐making [30]. Another point of evaluation concerns the need for quality‐control programs for standardized MRD assessment. In this sense, sample control, process control, and clarity of conclusion are key points. Innovative external quality‐control programs, which aim at monitoring the flow‐cytometry process for MM MRD testing, will allow for better inter‐laboratory alignment, providing valuable support to the application of flow‐MRD in routine clinical practice.

As for NGS, the method has been successfully implemented in all the centers participating in this MRD network, thanks to the extensive expertise in NGS in all centers, the robustness and reliability of the NGS method [31] and not least, the efficient management of samples and reagent distribution throughout this network.

The overall aim of the three NGS QC rounds was to test the reproducibility of the method [32] between centers, both in the clonotype identification (QC1) and in MRD monitoring, starting either from DNA (QC2) or from aliquot of cells of MRD mock samples (QC3). Overall, clonotype(s) was successfully detected by all centers; however, some concerns emerged regarding the selection of the most appropriate clonotype(s) to track, particularly when multiple options were suitable, including IGHFR1, FR2, FR3 and IGK. Indeed, clonotype selection can significantly impact subsequent MRD analysis, potentially leading to false‐negative or false‐positive results. Therefore, the selected sequence should be both frequent and unique, raising the challenge of defining its uniqueness. To address this, comparing the selected sequence with large databases of IG sequences (e.g., IMGT and BlastN) would be valuable, although no established guidelines currently exist to support this process.

In addition, other issues emerged from this first QC round: (1) the employment of either one or more clonotype(s) to monitor MRD to improve the overall sensitivity; (2) the optimization of the NGS workflow for ID clonotype(s) identification, which can be approached either by initially sequencing FR1 and IGK, followed by other regions in case of failure, or by sequencing FR3‐IGK or the combination of FR1‐FR2‐FR3‐IGK; (3) the use of positive and negative controls, which need to be optimized throughout different phases of NGS experiments (amplification, sequencing, and clonality assessment).

Notably, QC1 was performed on samples selected for their high quality, which may not reflect typical daily conditions, as sample hemodilution [33] or variations in DNA quality and quantity may occur [34]. Therefore, future QC rounds should also include technically critical samples, such as hypocellular BM. Similarly, QC2 and QC3 results, concerning MRD monitoring by NGS, demonstrated that the method can be employed successfully across different centers. In fact, by sharing at least two MRD mock samples for each patient included in the study, high concordance (81% and 91%) was observed. The main discrepancies were attributed to differences in the starting material. To avoid the low DNA input observed in some reactions, which led to reduced read depth and, consequently, lower sensitivity, a larger amount of DNA should be provided if samples are shared for future QC rounds.

The sensitivity of MRD measurement was the most important issue in QC2 and QC3. Although in these initial QC rounds it was decided to test high levels of MRD (i.e., 10^−3^ and 10^−4^) aiming at verifying the inter‐center reproducibility under more favorable conditions, the minimum sensitivity of 10^−5^ required to support clinical decisions in MM has been reached by the majority of the participants, paving the way for future quality control rounds, including more diluted MRD mock sample dilutions, approaching the method's LOD of 10^−5^ and 10^−6^. Pushing the method to its analytical limit, by increasing the DNA input and the total number of sequencing reads, should allow for confirmation of inter‐center concordance.

In conclusion, the NGS QC rounds showed that the molecular approach is feasible and reliable across centers (Table 4). Discussions within the network have been highly productive, emphasizing the importance of sharing experiences to reach a consensus and ultimately establishing shared guidelines for NGS use in daily practice [35, 36, 37].

**TABLE 4:** Level of concordance of NGS results reached across QC1‐QC2‐QC3 between centres.

NGS	Networks' quality control	N. concordant results	N. discordant results	% Concordance between centers
QC1	Clonotype identification	32	0	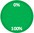
	Choice of the trackable clonotype	3	1	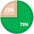
QC2	Clonal frequency	16	4	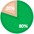
	Level of sensitivity 10^−5^	19	1	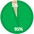
QC3	Clonal frequency	21	2	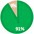
	Level of sensitivity 10^−5^	19	4	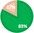

*Note:* Level of concordance. Number of concordant and discordant results obtained in each network quality control. The denominators employed to calculate the percentage of concordance varied across the different tasks. For QC1 (clonotype identification) the percentage of concordance has been calculated on the total number of evaluable reactions for each centre (32, excluding the NTC); for QC1 (choice of the trackable clonotype), the percentage of concordance has been calculated based on the reached consensus for each identified clonotype between the total of centres (4); for QC2 and QC3 (clonal frequency and level of sensitivity 10^−5^) the percentage of concordance has been calculated on the total of evaluable reactions for each centre (20 for QC2; 23 for QC3).

Overall, this networking experience has laid the foundation for strengthening collaboration among Italian centers, providing a preliminary framework for potential national group aiming to incorporate MRD analysis into the routine management of MM patients. Our experience has some limitations, including the limited number of tests and laboratories involved and the lack of heterogeneity of conditions that may occur in daily practice, but was intended as a preliminary step, for further extension to a wider audience. Importantly, the project was not designed to compare the two BM‐based strategies (i.e., NGF and NGS), as previous data have already demonstrated their interchangeability and highlighted the technical pros and cons of each approach. Instead, the project's goal was to identify procedures in order to develop harmonized protocols that can be easily implemented to ensure consistent results across Italian laboratories, with the ultimate aim of supporting the expansion of the project to a larger network of centers.

## Author Contributions

**Stefania Oliva:** conceptualization (equal), methodology (equal), project administration (equal), resources (equal), writing – original draft (equal), writing – review and editing (equal). **Marina Martello:** investigation (equal), methodology (equal), project administration (equal), writing – original draft (equal), writing – review and editing (equal). **Elona Saraci:** investigation (equal), methodology (equal), writing – original draft (equal), writing – review and editing (equal). **Silvia Armuzzi:** investigation (equal), writing – review and editing (equal). **Vincenza Solli:** formal analysis (equal), writing – review and editing (equal). **Simona Barbato:** project administration (equal), writing – original draft (equal), writing – review and editing (equal). **Angelo Belotti:** investigation (equal), writing – review and editing (equal). **Niccolò Bolli:** funding acquisition (equal), investigation (equal), writing – review and editing (equal). **Clara Bono:** investigation (equal), writing – review and editing (equal). **Francesco Buccisano:** investigation (equal), writing – review and editing (equal). **Marco Chiarini:** investigation (equal), writing – review and editing (equal). **Maria Antonietta Irno Consalvo:** investigation (equal), writing – review and editing (equal). **Iole Cordone:** investigation (equal), writing – review and editing (equal). **Daniela Drandi:** investigation (equal), writing – review and editing (equal). **Sara Galimberti:** investigation (equal), writing – review and editing (equal). **Elisa Genuardi:** investigation (equal), writing – review and editing (equal). **Viviana Giustini:** investigation (equal), writing – review and editing (equal). **Francesca Guerrini:** investigation (equal), writing – review and editing (equal). **Marta Lionetti:** investigation (equal), writing – review and editing (equal). **Akihiro Maheda:** investigation (equal), writing – review and editing (equal). **Sara Marino:** investigation (equal), writing – review and editing (equal). **Serena Masi:** investigation (equal), writing – review and editing (equal). **Antonio Matera:** investigation (equal), writing – review and editing (equal). **Andrea Mengarelli:** investigation (equal), writing – review and editing (equal). **Nunziatina Laura Parrinello:** investigation (equal), writing – review and editing (equal). **Aldo Maria Roccaro:** investigation (equal), writing – review and editing (equal). **Alessandra Romano:** investigation (equal), writing – review and editing (equal). **Giovanni Rossi:** investigation (equal), writing – review and editing (equal). **Barbara Taurisano:** investigation (equal), writing – review and editing (equal). **Alessia Tonini:** investigation (equal), writing – review and editing (equal). **Valentina Trimarco:** investigation (equal), writing – review and editing (equal). **Anna Maria Triolo:** investigation (equal), writing – review and editing (equal). **Ilaria Vigliotta:** investigation (equal), methodology (equal), writing – review and editing (equal). **Renato Zambello:** investigation (equal), writing – review and editing (equal). **Benedetto Bruno:** investigation (equal), resources (equal), writing – review and editing (equal). **Michele Cavo:** resources (equal), supervision (equal), writing – review and editing (equal). **Elena Zamagni:** conceptualization (equal), funding acquisition (equal), investigation (equal), resources (equal), supervision (equal), writing – review and editing (equal). **Carolina Terragna:** conceptualization (equal), investigation (equal), methodology (equal), project administration (equal), resources (equal), supervision (equal), writing – original draft (equal), writing – review and editing (equal).

## Funding

This work has been supported by the Ministry of Health (RC‐2025‐2797269).

## Ethics Statement

The study was approved by the Comitato Etico Area Vasta (CE‐AVEC) and by the local Ethics Committee at each participating center. The study was conducted in compliance with the Declaration of Helsinki and Good Clinical Practice guidelines. All patients provided written informed consent before entering the study.

## Conflicts of Interest

S.O. has served in advisory board and has been consultant for: Amgen, Celgene/Bristol‐Myers Squibb Janssen, Sanofi, Pfizer, Abbvie, Adaptive Biotechnologies, Takeda; M.M. has received honoraria from Sanofi, GSK and Instrumentation Laboratory SpA‐Werfen; A.B. has participated in Advisory Board for Amgen, GSK, Pfizer, Sanofi, Menarini‐Stemline, Janssen; N.B. has received honoraria for Amgen, GSK, Janssen, Jazz, Oncopeptides, Pfizer, Sanofi, Takeda; F.B. has received honoraria for consulting/Advisory Boards from Jazz Pharmaceuticals, Laboratoires Delbert, Novartis, and as a Speaker from Astellas, Bristol‐Myers Squibb, Janssen‐Cilag, Servier; A.M.R. has received research funding from AstraZeneca and honoraria from Abbvie, Amgen, Beigene, Celgene, Janssen, Roche, Takeda; R.Z. has served in advisory boards for Janssen, Sanofi, GSK, Oncopeptides, Menarini, Pfizer; B.B. has received honoraria from GlaxoSmithKline, Sanofi, Roche, Janssen, Astrazeneca, Beone, Novartis, and has served on the advisory boards for GlaxoSmithKline, Sanofi, and Bristol‐Myers Squibb; M.C. has served in a consulting/advisory role for and has received honoraria from Amgen, AbbVie, Bristol‐Myers Squibb, Celgene, GlaxoSmithKline, Janssen, Menarini‐Stemline, Sanofi, and Karyopharm Therapeutics; E.Z. has received honoraria and has served in advisory role for Janssen, Bristol‐Myers Squibb, Sanofi, Amgen, GlaxoSmithKline, Pfizer, Oncopeptides, Menarini‐Stemline; C.T. has received honoraria from Sanofi, GSK, Janssen and Instrumentation Laboratory SpA‐Werfen. E.S., S.A., V.S., S.B., C.B., M.C., M.A.I.C., I.C., D.D., S.G., E.G., V.G., F.G., M.L., A.M., S.M., S.M., A.M., A.M., N.L.P., A.R., G.R., B.T., A.T., V.T., A.M.T., and I.V. declare no potential conflicts of interest.

## Supporting information


**Appendix S1:** cam471678‐sup‐0001‐AppendixS1.docx.

## Data Availability

Data supporting the findings of this study are available via Zenodo platform 10.5281/zenodo.15846711.
