# Supplementary material for: Next Generation Flow and Next Generation Sequencing for Measurable Residual Disease Assessment in Multiple Myeloma Patients: A Real‐Life Italian Multicenter Harmonization Experience
Source: Cancer Med. 2026 Mar 17;15(3):e71678. doi: 10.1002/cam4.71678 (PMC13093631; doi:10.1002/cam4.71678)
Supplement: Supplementary file 1 — Appendix S1: cam471678‐sup‐0001‐AppendixS1.docx. [file CAM4-15-e71678-s001.docx]

**Supplementary**

**1- Supplementary Methods**

**1.1 - Sample processing and statistical methods on NGF results**

MRD was evaluated according to NGF-EuroFlow protocol ([www.euroflow.org](http://www.euroflow.org)). A bulk lysis of red blood cells was performed before staining. For PCs identification, a two-tube, 8-color antibody panel was used: **Tube 1** (CD27, CD138, CD38, CD56, CD45, CD19, CD117, CD81) and **Tube 2** (CD27, CD138, CD38, CD56, CD45, CD19, cytoplasmic κ and λ light chains)^5^.

Cell acquisition was performed using BD FACS Canto II, BD FACS Lyric, BC Navios, and BC DxFlex flow-cytometers, following the standardization of instrument settings according to the EuroFlow Standard-Operating-Protocol (SOP) for setup and compensation. Our goal was to acquire ≥3.000.000 events per tube to achieve a sensitivity of at least 1×10⁻⁵. Samples were considered MRD-positive if ≥20 malignant PCs (mPCs) were detected. The limit of detection (LOD) and limit of quantitation (LOQ) were defined as ≥20 and ≥50 mPCs per total nucleated cells acquired, respectively. Infinicyt™ software (Cytognos SL, Salamanca, Spain) was used to analyze flow-cytometry data. In L2 and L6 laboratories, where this software was unavailable, Kaluza 2.1 and BD FACS Diva were used, respectively. The Intraclass Correlation Coefficient (ICC) was employed to quantify the degree of correlation and agreement between measurements, ensuring a robust reliability assessment. Additionally, the standard deviation (SD) and coefficient of variation (CV) were calculated to characterize the extent of variability within the dataset.

**1.2 - Sample preparation for NGS experiments**

BM aspirates were collected with a 20 mL syringe, containing 1 ml of EDTA as anticoagulant. Samples were then shared with participating centers, as described in **Supplementary Fig. 1**. For each NDMM patient, BM samples were evaluated by flow-cytometry before PCs enrichment, to determine whether the samples had sufficient cell numbers to proceed. Then, bulk lysis was performed on BM and, prior to immunomagnetic cell staining and enrichment, cells were enumerated, as described elsewhere^9^. Once the isolated CD138+ PCs were obtained, the purity of the cell fraction was evaluated by flow-cytometry. An aliquot of CD138+ cells was then used for diagnostic DNA isolation (1x10^6^ cells), and the remaining cells were counted and used to prepare MRD mock samples. For this purpose, PCs were diluted in white blood nuclear cells derived from a pool of healthy donors, to prepare mock samples for use as 10^-3^ and as 10^-4^ MRD surrogates.

For diagnostic samples, each center received 500 ng of DNA (i.e., sufficient to perform the clonality screening experiments, 70 ng each), along with 6.5 µg of DNA for each mock sample (i.e., sufficient to test 3 replicates for each MRD measurement), and 100x10^6^ cells of MRD mock samples for MRD analyses. Libraries were prepared according to the shared protocols. For each diagnostic samples, 4 different assays (IGHFR1, IGHFR2, IGHFR3 and IGK) were tested in a unique V2 500 Miseq run (2x250 paired-end); then, the sequence identified as clonotypic was chosen to be tracked in the MRD mock samples, by running three replicates for each sample, for a total of three V3 600 Miseq runs (2x300 paired-end). For each experiment, a negative, a positive, and non-template controls were included. FASTq files were processed according to the Invivoscribe pipeline (<https://invivoscribe.com/>).

**2- Supplementary tables**

**Supplementary Table 1: QC1-2-3 sequencing results**

C1: Bologna-IRCCS AOUBO, C2: Milano-Fondazione IRCCS Ca’Granda, C3: Pisa-AOU, C4: Torino-UniTo.

QC1-2-3: quality control 1-2-3

**Supplementary Table 2: Variation in the measurement of bone marrow populations and MM MRD related parameters between laboratories (stage 1)**

CV: coefficient of variation; LOD: limit of detection; LOQ: limit of quantitation; MM: multiple myeloma; MRD: minimal residual disease; SD: standard deviation.

**Supplementary table 3: Variation in the measurement of bone marrow populations and MM MRD related parameters between laboratories (stage 2)**

CV: coefficient of variation; LOD: limit of detection; LOQ: limit of quantitation; MM: multiple myeloma; MRD: minimal residual disease; SD: standard deviation; WBC: white blood cells.

**Supplementary table 4: Criteria for clonotype selection in QC1, example of selection from C1**

| **QC1** | | **IMGT/V-QUEST and BLAST INFORMATION** | | | |
| --- | --- | --- | --- | --- | --- |
| **PT** | **ASSAY** | **V identity %** | **J identity %** | **Junction** | **Blast score** |
| **PT_1** | IGHFR1 | 88,07 | 83,87 | N1=15nt; N2= 9nt | 303 |
|  | IGHFR2 | 89,16 | 83,87 | N1=15nt; N2= 9nt | 228 |
|  | IGHFR3 | 90,24 | 83,87 | N1=15nt; N2= 9nt | 76 |
|  | IGK | 78,70 | 94,74 | N1= 0nt; N2= nt | 446 |
| **PT_2** | IGHFR1 | 90,41 | 75,75 | N1=0nt; N2 = 9nt | 319 |
|  | IGHFR2 | 88,55 | 75,76 | N1= 0nt; N2 = 9nt | 231 |
|  | IGHFR3 | 90 | 75,76 | N1= 0nt; N2 = 9nt | 76 |
|  | IGK | 96,00 | 90,91 | N1= 4nt; N2 = 0 nt | 383 |
| **PT_3** | IGHFR1 | 93,36 | 91,18 | N1= 5nt, N2=5nt | 364 |
|  | IGHFR2 | 93,87 | 91,18 | N1= 5nt, N2=5nt | 300 |
|  | IGHFR3 | 95 | 91,18 | N1= 5nt, N2=5nt | 75 |
|  | IGK_KDE | 100,00 | not found | not found | 421 |
| **PT_4** | IGHFR1 | 94,06 | 97,20 | N1= 2 nt; N2= 7 nt | 387 |
|  | IGHFR2 | 93,98 | 97,22 | N1= 2 nt; N2= 7 nt | 296 |
|  | IGHFR3 | POLYCLONAL | POLYCLONAL | POLYCLONAL | POLYCLONAL |
|  | IGK | not found | not found | not found | 300 |
| **PT_5** | IGHFR1 | 77,67 | 87,5 | N1= 17 nt; N2= 2 nt | 300 |
|  | IGHFR2 | POLYCLONAL | POLYCLONAL | POLYCLONAL | POLYCLONAL |
|  | IGHFR3 | 88,89 | 87,5 | N1= 17 nt; N2= 2 nt | 108 |
|  | IGK | ne | ne | ne | ne |
| **PT_6** | IGHFR1 | 85,91 | 83,87 | N1= 0 nt; N2= 4 nt | 294 |
|  | IGHFR2 | POLYCLONAL | POLYCLONAL | POLYCLONAL | POLYCLONAL |
|  | IGHFR3 | 95,00 | 83,87 | N1= 0 nt; N2= 4 nt | 89,1 |
|  | IGK | 100,00 | 71,43 | N1= 0 nt; N2= 4 nt | 377 |
| **PT_7** | IGHFR1 | 87,56 | 74,29 | N1= 4 nt; N2=11 nt | 302 |
|  | IGHFR2 | 88,73 | 74,29 | N1= 4 nt; N2= 11 nt | 231 |
|  | IGHFR3 | ne | ne | ne | ne |
|  | IGK | 95,75 | 100 | N1= 0 nt; N2= nt | 289 |
| **PT_8** | IGHFR1 | 88,13 | 88,87 | N1=15 nt; N2=1 nt | 283 |
|  | IGHFR2 | 87,35 | 83,87 | N1= 4 nt; N2= 11 nt | 211 |
|  | IGHFR3 | 85,19 | 76,09 | N1=14 nt; N2= 20 nt | 67 |
|  | IGK | 95,45 | 94,74 | N1= 0 nt; N2= nt | 289 |

The table summarized the criteria that were employed for the selection of a specific and precise clonotype for MRD monitoring. This is an example from center C1. Rearrangements are selected to be clonotype based on the % of V and J identifIES (the lowest the percentage; the most original is the sequence), the total number of nucleotides in the insertion (the more, the better), and the BLAST score (inversely proportional to the originality of the clone, but it must still be evaluated as a whole together with the other parameters).V=Variable segment; J=Joining segments; nt=nucleotides; N1= Number of nucleotides of the N1-REGION; N2= Number of N nucleotides of the N2-REGION; not found=no alignment is produced on IMGT/V-QUEST tool; ne=not evaluable; POLYCLONAL=not evaluable rearrangement was found.

In yellow, the targets selected for the MRD monitoring are highlighted.

**3- Supplementary Figures**

**Supplementary Fig. 1: Sample preparation schema for NGS experiments included in the QC1, QC2 and QC3.**


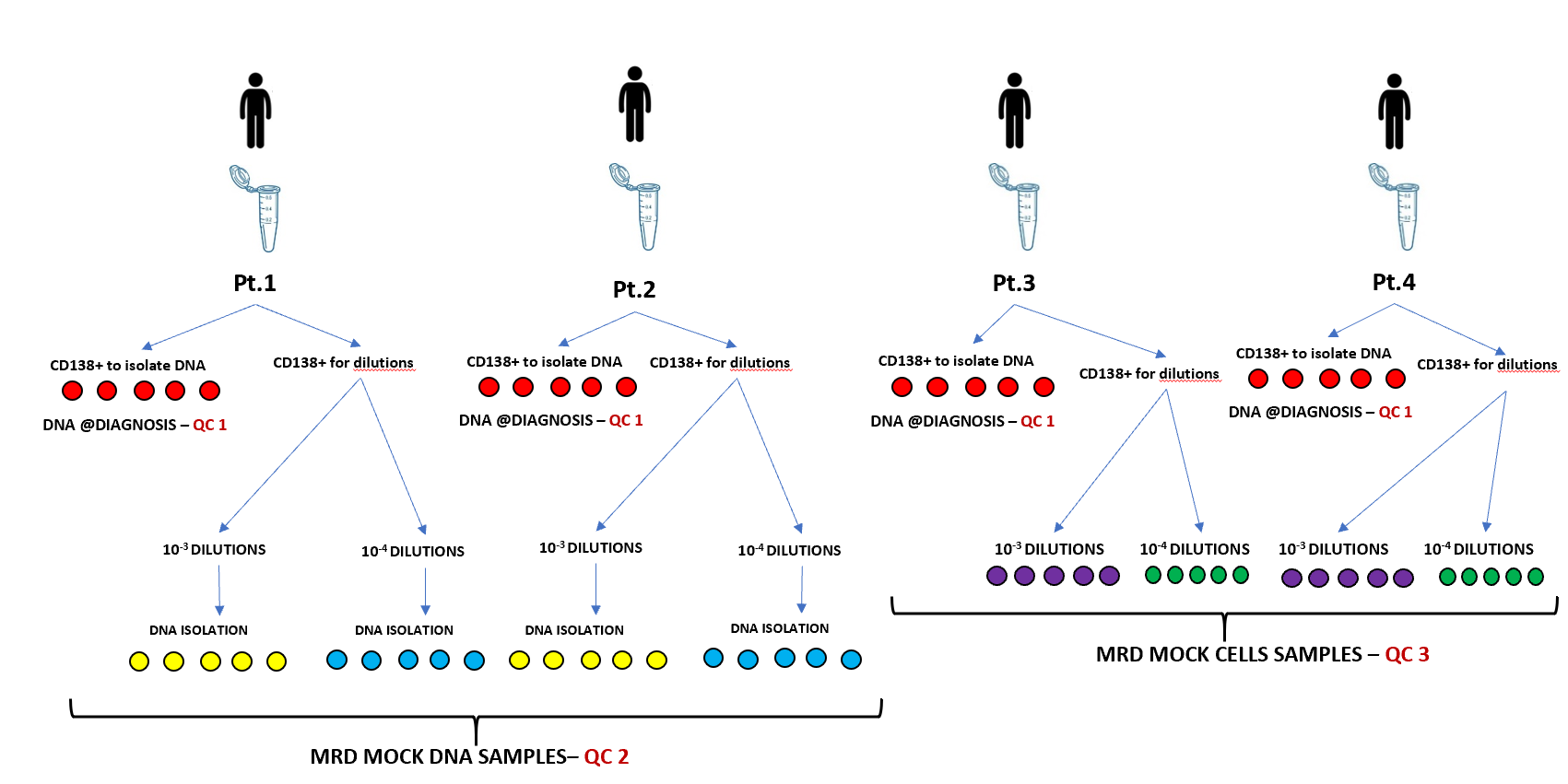


**Abbreviations:** QC= quality control; MRD= minimal residual disease; Pt= patient.

**Supplementary Fig. 2 QC-1 ID clonotype identification by NGS. Concordance rate from the first NGS experiment performed by the network of 4 laboratories (C1, C2, C3, C4)**

Collectively, QC1 demonstrated 100% concordant (green checkmark) identification of rearrangements by NGS across centers, considering only successful experiments. Major discussions (head with gears) emerged around selecting the trackable clonotype, especially when multiple regions (such as IGHFR1, FR2, FR3, and IGK) were suitable for this purpose. Due to the lack of clear guidelines for clonotype selection to monitor MRD, agreement among centers varies widely (from 50% to 100%), highlighting the need for standardized, shared recommendations.

**
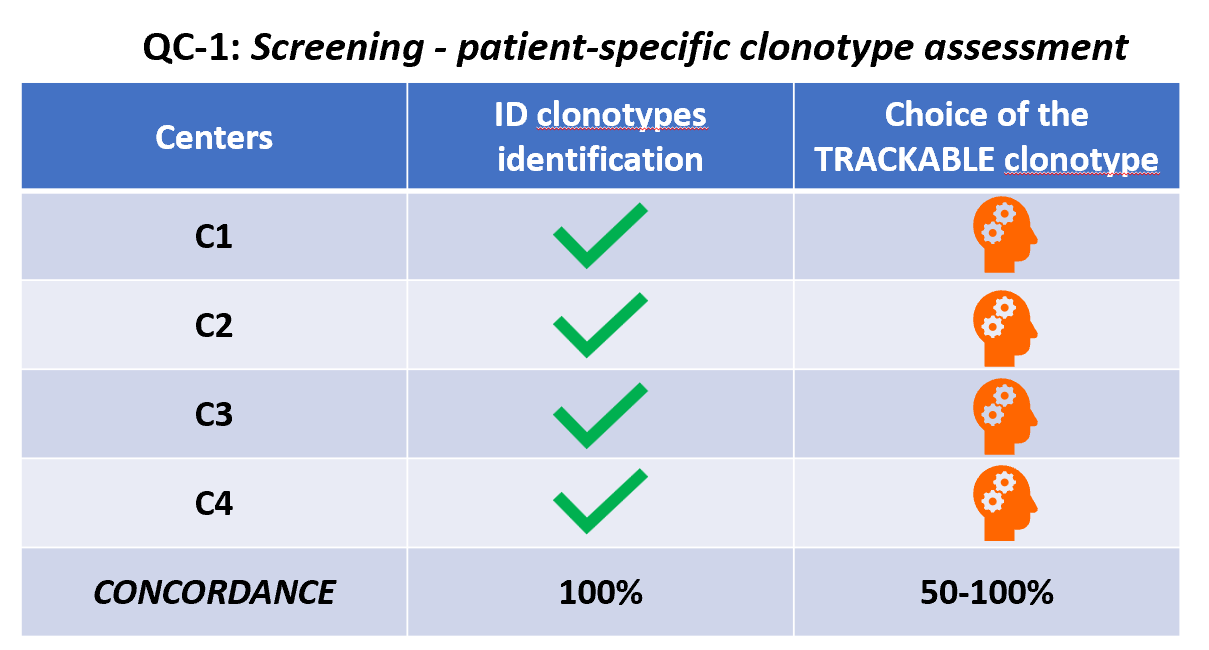
**

C1: Bologna-IRCCS AOUBO, C2: Milano-Fondazione IRCCS Ca’Granda, C3: Pisa-AOU, C4: Torino-UniTo.

NGS: Next-Generation Sequencing; QC1: quality control 1
